# Disentangling sequential from hierarchical learning in Artificial Grammar Learning: Evidence from a modified Simon Task

**DOI:** 10.1371/journal.pone.0232687

**Published:** 2020-05-14

**Authors:** Maria Vender, Diego Gabriel Krivochen, Arianna Compostella, Beth Phillips, Denis Delfitto, Douglas Saddy

**Affiliations:** 1 Department of Cultures and Civilizations, University of Verona, Verona, Italy; 2 Centre for Integrative Neuroscience and Neurodynamics, University of Reading, Reading, United Kingdom; Tohoku University, JAPAN

## Abstract

In this paper we probe the interaction between sequential and hierarchical learning by investigating implicit learning in a group of school-aged children. We administered a serial reaction time task, in the form of a modified Simon Task in which the stimuli were organised following the rules of two distinct artificial grammars, specifically Lindenmayer systems: the Fibonacci grammar (Fib) and the Skip grammar (a modification of the former). The choice of grammars is determined by the goal of this study, which is to investigate how sensitivity to structure emerges in the course of exposure to an input whose surface transitional properties (by hypothesis) bootstrap structure. The studies conducted to date have been mainly designed to investigate low-level superficial regularities, learnable in purely statistical terms, whereas hierarchical learning has not been effectively investigated yet. The possibility to directly pinpoint the interplay between sequential and hierarchical learning is instead at the core of our study: we presented children with two grammars, Fib and Skip, which share the same transitional regularities, thus providing identical opportunities for sequential learning, while crucially differing in their hierarchical structure. More particularly, there are specific points in the sequence (*k-*points), which, despite giving rise to the same transitional regularities in the two grammars, support hierarchical reconstruction in Fib but not in Skip. In our protocol, children were simply asked to perform a traditional Simon Task, and they were completely unaware of the real purposes of the task. Results indicate that sequential learning occurred in both grammars, as shown by the decrease in reaction times throughout the task, while differences were found in the sensitivity to *k-*points: these, we contend, play a role in hierarchical reconstruction in Fib, whereas they are devoid of structural significance in Skip. More particularly, we found that children were faster in correspondence to *k-*points in sequences produced by Fib, thus providing an entirely new kind of evidence for the hypothesis that implicit learning involves an early activation of strategies of hierarchical reconstruction, based on a straightforward interplay with the statistically-based computation of transitional regularities on the sequences of symbols.

## 1. Introduction

Artificial grammar learning (AGL) is an experimental paradigm employed to investigate how sequences of symbols produced by a system are learnt, as well as to assess implicit learning, i.e. learning that occurs incidentally, without explicit awareness of what has been learnt. An artificial grammar is characterised by a finite alphabet of symbols and a finite set of rules, which, applying to these symbols, produce specific strings. In this paper, we intend to study the interplay between sequential and hierarchical learning, aiming to disentangle the two processes. We start by providing a short introduction to AGL, discussing the most important findings that have been reported using this methodology, as well as their major weaknesses. We then focus on the technical aspects of the Lindenmayer systems, and in particular of the two grammars that we employed in our experimental protocol, the Fibonacci grammar and its non-trivial modification Skip. After laying out our research questions and predictions, we proceed with a detailed presentation of the proposed experimental design. Finally, we discuss our experimental results, elucidating some of the non-trivial theoretical consequences that these results suggest.

As a starting point, consider the fact—by now confirmed by a large number of studies—that participants exposed to strings produced by artificial grammars show evidence of developing a rule-based representation of the grammar, being able, for instance, to discriminate between grammatical and ungrammatical strings (strings that are produced by a grammar and strings which are not, respectively), although their knowledge generally remains implicit [1]. These learning effects have been reported in studies conducted on babies who were able to detect patterns and distributional regularities of artificial grammars as early as at the age of 8 months [2, 3].

Considering that processing a language requires the subject to analyse or “parse” a sequence of sounds or symbols, to detect regularities and to extend learnt regularities to new sequences, it is not surprising that implicit learning abilities have been found significantly correlated to linguistic performance [4]. This was further confirmed by neurolinguistic evidence, showing that syntactic processing of linguistic stimuli and sequential learning of structured sequence patterns share the same neural mechanisms (see [5] for an ERP study).

AGL studies can thus provide a privileged window in the investigation of how humans process external stimuli and perform higher-order cognitive functions, especially for what concerns language processing [6]. The great advantage of artificial versus natural grammars lies in the possibility to work in controlled settings using language-independent rules and non-linguistic stimuli, devoid of semantic content: first, participants taking part in an AGL task have never been exposed to strings produced by that grammar before, which allows a purer inspection of how the learning process develops, permitting the study of implicit learning of grammatical rules without the interference of semantic and pragmatic aspects that cannot be inhibited during natural language tasks [7]. Second, using an artificial grammar gives researchers the possibility to fine-tune a paradigm on which they have full control, as the grammar is completely determined by the mathematical rules characterising it, and to directly choose the patterns to study, thus allowing us to assess precise and testable hypotheses. There are two fundamental aspects that should be discussed when considering implicit learning of artificial grammars: on the one side, the type of task that is used, and on the other side, the type of grammar that is adopted.

As for the first aspect, the most widely-employed technique has the form of a grammaticality judgment task (see [8] for a pioneering work). In this paradigm, participants are first presented with the stimuli produced by the grammar and asked to memorise them. Upon completion of this learning phase, they are generally informed about the existence of a grammar and of specific rules according to which those stimuli had been arranged, without however being explicitly presented with the relevant rules. They are then shown a new set of stimuli and asked for grammaticality judgments on the basis of what they had learnt about the grammar from the items they had previously memorised. Typically, people display an above-chance performance, suggesting that implicit learning of the grammar took place, although this knowledge remains largely unconscious (see [1] and references therein for a detailed review of AGL studies). Although participants are unaware of the real purposes of the task, considering it just a memory test, its implicit nature is still questionable: implicit cognition should indeed be completely automatic and unconscious, without any guidance or cues during the learning process, as opposed to explicit cognition, in which instead a conscious effort is required to memorise and retrieve information during learning [1]. As observed by [9], to provide a grammaticality judgment, participants are forced to render their knowledge of the grammar somehow accessible and explicit processing is required if they are asked to perform a conscious comparison between memorised items and new ones, which can critically affect the implicitness of the task.

Implicitness remains preserved, instead, in serial reaction time (SRT) tasks, a different experimental technique which can be employed to track learning progresses of a structured sequence of stimuli [10]. In this paradigm, participants are typically exposed to a sequence of visual stimuli on a computer screen and they are asked to press some keys as fast and as accurately as possible. The sequence of items is determined by an artificial grammar, intertwined with random sets. In this case, participants remain completely unaware of the real purpose of the task for the whole experiment (they are told that the aim is that of investigating the effects of practice on motor performance) and, importantly, they are never informed that the sequence of stimuli follows the rules of an artificial grammar, nor asked for grammaticality judgments. Interestingly, a decrease in RTs is noticed as the task progresses in structured but not in random sequences, clearly indicating that participants become increasingly sensitive to the sequential structure of the material and providing evidence for a type of learning which, in this case, really qualifies as implicit [11,12].

The other major factor that should be addressed in implicit learning studies concerns the choice of the grammar to employ. By exploiting distinctive properties within and between formal grammars, AGL studies allow us to address the interplay between sequential and hierarchical learning and the debate concerning the nature of human language learning and processing. At this point, we need to introduce some basic notions of formal language theory, intended to highlight the relevance of our paradigm in connection to previous research. Technically, a grammar G is a set G = (Σ,V_N_, V_T_, δ), where:

- Σ is the input alphabet;
- V_N_ is a set of non-terminal symbols (which can appear at the left-hand side of the rules in (iv));
- V_T_ is a set of terminal symbols (which cannot appear at the left-hand side of rules in (iv));
- δ is a set of rules of the general form A → B, ‘rewrite A as B’ [13,14].

Rules apply sequentially (a modality known as a *traffic convention*), producing grammatical ‘strings’ (i.e. ‘words’ or ‘sentences’ [13,14]).

Until now, AGL studies have normally used grammars within the Chomsky Hierarchy [13], that is, grammars which conform to the canonical description above.

Though a study conducted by [15], for instance, seems to hint that the human parser is not sensitive to hierarchical syntactic structure, thus suggesting that linguistic processing has a purely sequential nature, subsequent results by [16], who replicated and extended their work, actually found evidence for hierarchical processing in human language. Similarly, while [17] showed that morphological processing is sensitive to statistically-based transitional probabilities, [18] reported evidence for rule learning mechanisms in derivational morphology. The AGL paradigm could thus be effectively employed to study the interplay between the two types of learning, thus shedding more light on this debate, even though it must be acknowledged that investigating hierarchical processing in AGL studies is not trivial, due to the characteristics of the grammars that are typically used. The majority of the AGL studies, indeed, employed strings produced by means of transition graphs which proceed step-wise from one node (or state) to the next and outputting one item at a time to produce a linear string of symbols [1,8]. However, such grammars lack the computational power to provide adequate structural representations for *all and only* grammatical sentences in human languages [19–23]. Therefore, AGL research to date has only been able to reliably investigate *sequential* learning abilities (based on transitions between symbols in a string), without directly addressing *hierarchical* aspects of grammatical processing.

Nevertheless, this latter issue has been specifically addressed by more recent studies which aimed at finding evidence for recursive structure learning by employing context-free grammars ([24]; see also [25] for an fMRI study). In a well-conceived protocol, [26] assessed implicit learning with four types of grammars producing *a*^*n*^*b*^*n*^ (i.e., an *n* number of *a*s followed by the same number of *b*s, for *a* and *b* terminals) patterns, featuring the distinction between left- and right-branching structures, as well as between centre-embedding and head-tail recursion (two types of distinctions which can be found in human languages) and showing that participants were able to learn even long-distance dependencies. However, using these grammars as a test for recursion cannot be considered a decisive proof for hierarchical learning, as the ability of discriminating grammatical from ungrammatical strings of the form *a*^*n*^*b*^*n*^ could be reduced to counting or subitising mechanisms more than to centre-embedding [27–29]. Furthermore, parallels cannot easily be drawn between processing of strings from the *a*^*n*^*b*^*n*^ grammar and centre-embedding in natural language, due to working memory constraints on parsing any more than a small number of hierarchically embedded clauses. These protocols aim at showing that subjects can distinguish strings produced by a number of phrase structure grammars, but, crucially, the connection between the superficial properties of these strings and the mechanisms whereby subjects develop a representation of a grammar is not addressed. This is so because in experimental conditions a finite number of finite strings can be used and for any finite value of *n* there is a finite-state parsing of *a*^*n*^*b*^*n*^ [30]; strict context-freeness arises only *for all* values of *n*. In these cases, neglecting the analysis of superficial regularities in a string (including information about transition probabilities between adjacent and non-adjacent symbols and sequences thereof, statistical frequencies and distributions…) would lead to incorrect claims about the characteristics of a grammar consistent with producing those strings (for example, that it can infer hierarchical structure when a linear parsing mechanism would be sufficient). From this perspective, an important feature of the present paradigm is that the interplay between superficial regularities and hierarchical properties of the language under consideration is at the core of the design: the choice of Fib as our target grammar is due precisely to the fact that a superficial transitional property maps onto an abstract, hierarchical property of the structure (see the Discussion); the goal of the present study is to assess human sensitivity to abstract structural properties as distinct from (but related to, and even emergent from) superficial linear regularities.

In summary, although they must be credited for providing interesting results, most of the existing studies assessing implicit learning face two potential problems: (i) in terms of cognitive underpinnings, asking for grammaticality judgments undermines the implicitness of the task; (ii) in addressing linguistic concerns, canonical grammars do not really exploit the potentials of AGL, since they are in fact not best suited to easily disentangle sequential and hierarchical learning.

In this study we address both issues, by proposing a SRT task, which guarantees, as discussed above, real implicitness of learning. We employed a modified Simon task in which the sequence of the stimuli is arranged following the rules of a noncanonical grammar, the Fibonacci grammar, which arguably constitutes an optimal tool in order to assess both sequential and hierarchical learning, as will be discussed in some detail in the following sections.

### 1.1. Studying AGL with a non-canonical grammar: The Fibonacci grammar

The Fibonacci grammar (Fib henceforth) is an asymmetric Lindenmayer system ([31]; see [32] for technical details, definitions, and a discussion of symmetry/asymmetry in L-systems [33, 34]). Fib, is defined by the alphabet Σ = {0, 1} and the following rewriting rules:

(1) 0 → 1
  1 → 0 1

Rules operate over symbols which are to be found in an input sequence; the first rule establishes that every instance of [0] in a sequence *s*_*n*_ must be rewritten as [1] at *s*_*n+1*_, while according to the second rule every instance of [1] in *s*_*n*_ is to be replaced by [1] at *s*_*n+1*_: the transition between *s*_*n*_ and *s*_*n+1*_will be called a *derivational step* [35].

**Fig 1:**
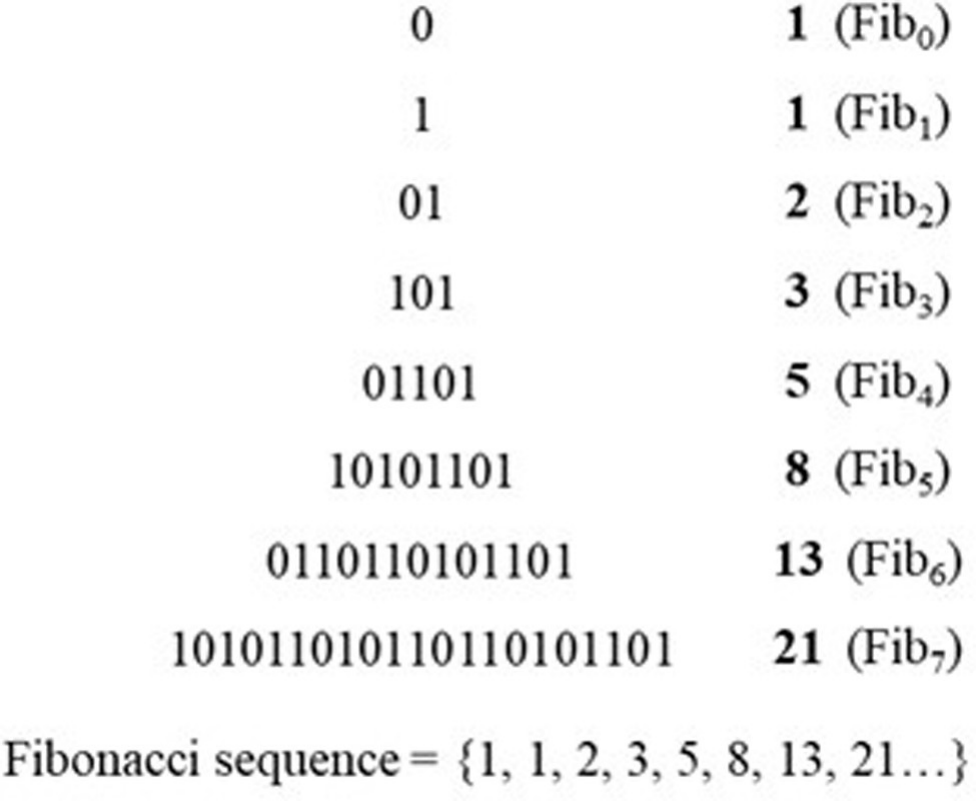
Graphical representation of a sequence produced by the Fibonacci grammar, where Fib_n_ indicates each generation of the grammar.

Applying these two rules produces sequences of symbols which can be represented as in Fig 1.

As can be noticed, the number of symbols of each generation *n* (notated Fib_n_) corresponds to the Fibonacci sequence, to which the grammar owes its name; the distribution of [0]s and [1]s in each string, starting respectively from generations Fib_2_ and Fib_1_, follows this sequence too.

Fib is a simple deterministic recursive rewrite system [31,33,34], with some peculiar properties representing fundamental differences from the canonical grammars discussed above. First, in classical L-systems (as discussed, e.g., in [33]) there is no distinction between non-terminals and terminals, which means that every symbol can (and thus must) be rewritten. Second, all rules that may apply do so *simultaneously*: L-grammars feature no Traffic Convention.

**Fig 2:**
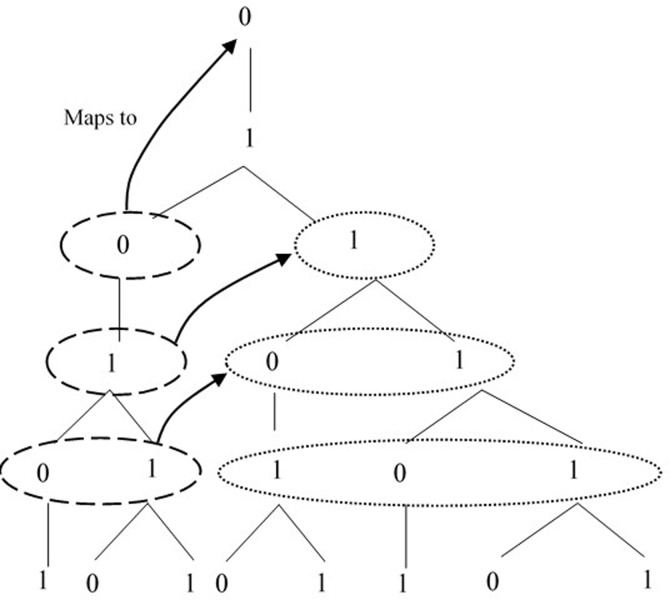
Representation of self-similarity.

Very informally, the asymmetry we mention pertains to the growth pattern of the grammar; developing the tree will reveal a ‘right-branching’ pattern rather than a balanced, symmetrical growth (as opposed to a grammar like Thue-Morse: 0 → 1 0; 1 → 0 1). More concretely, all [0]s and all [1]s that form *s*_*n*_ are rewritten at the same time to form *s*_*n+1*_. This implies a fundamental departure from sequential rule systems [14,35–37]. The third fundamental property of the L-systems is *self-similarity*: any structure-sensitive pattern found in the derivation can be mapped to an earlier stage of the derivation, at every scale [38]. The most basic illustration of *self-similarity* (because it builds on the recurrence relation Fib_n_ = Fib_n-1_ + Fib_n-2_ for any *n*th term of the sequence that defines the Fibonacci series) is shown in Fig 2.

The properties highlighted above pertain to the structural descriptions produced by L-grammars and the process whereby those structural descriptions come to be. Apart from these unique properties, L-grammars share many of the superficial aspects that characterise grammars in the Chomsky Hierarchy [13, 39]: they have an alphabet, a set of states (in our case, {1, 0}), and transition functions from one state to another (e.g., 0 is a state and so is 1; the rules 0 → 1 and 1 → 01 are transitions between states).

At this point, we need to consider another difference between L-systems and grammars in the Chomsky Hierarchy. Because L-systems do not have a distinction between terminal nodes and non-terminal nodes, derivations in classical L-systems (as defined in [33]) never halt since at no point there is a string of symbols which cannot be further rewritten. Each application of the rules to a string yields a new string, which is referred to as a *generation* of the grammar. The point to consider here is that there are some transitional properties of the Fib grammar at the superficial level which justify our choice as a target grammar. Within each Fib generation, we find the following transition regularities, which we will refer to as the Three Laws (see also [40]):

(2) (a) First Law: A [0] is always followed by a [1], i.e. 00 is not produced by the grammar (henceforth, it is ‘ungrammatical’).
  (b) Second Law: Two [1] are always followed by a [0], i.e. 111 is ungrammatical.
  (c) Third Law: A single [1] can be followed by either a [0] or a [1], i.e., [1] does not provide enough information for a parser to determine what symbol will come next. We will refer to this as an ‘ambiguity’.

Relying only on string-based statistical probabilities, Fib strings contain both points which can be totally predictable based on the regularities in (2a-b) (*deterministic points*), and points whose distribution is governed by (2c); the fact that there is more than a single possible continuation for the sequence [01**?**] motivates the denomination *non-deterministic points*. In other words, it is quite easy to predict that, given a [0], a [1] will follow (as per the First Law). More difficult to learn, instead, is that after a sequence of two [1]s, a [0] will follow (2b): this regularity plausibly requires additional resources, since participants need to store two items in memory, considering not only the immediate predecessor of the given item, but also the preceding one. Nevertheless, the [0] occurring after the bigram [11] is completely predictable.

For our purposes, the most interesting case is (2c): although a [1] is locally ambiguous, it should be noted that this ambiguity pertains only to left-to-right transition probabilities, and thus to sequential learning.

**Fig 3:**
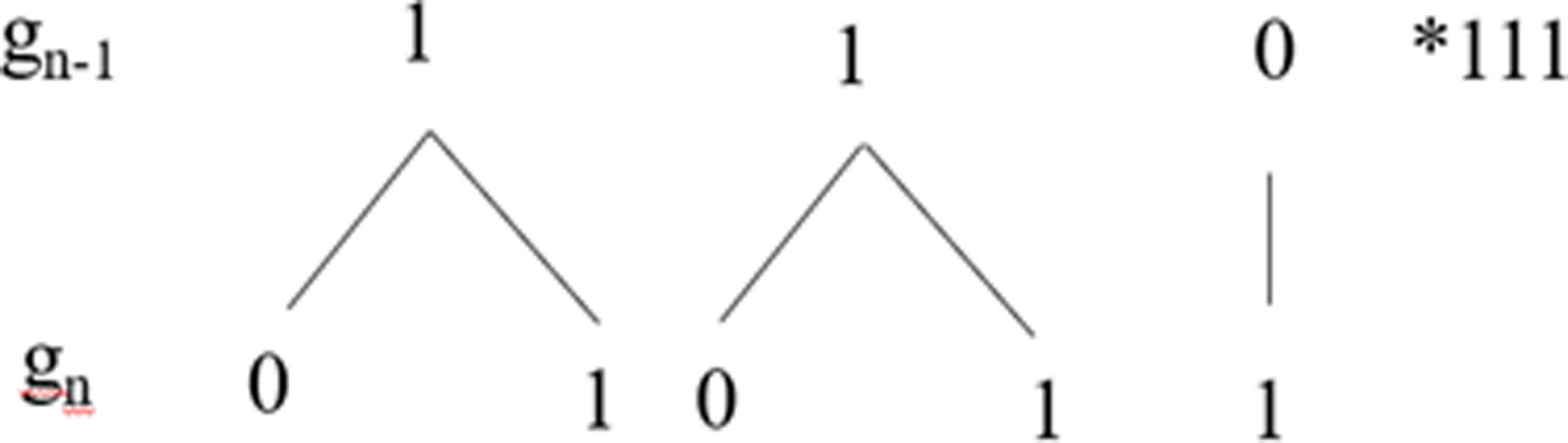
Disambiguation of the sequence [0101].

In fact, although Fib strings may look like a simple sequence of [0]s and [1]s, we aim at proving that humans in fact apply structure-sensitive parsing strategies to the Fib derivation on the basis of superficial information available in the string, which is used to reinforce structural hypotheses: these rest on the reliable availability of specific information at the string level, which we have presented here as the Three Laws *and* the way in which there is a homomorphic mapping between superficial and structural properties of the grammar (see the Discussion section). As a consequence, although the Third Law states that [1] is ambiguous, in the sense that both [10] and [11] are grammatical, it should be noticed that this holds only if [1] is considered independently of the hierarchical relations in which it participates. An important derivational property of Fib is that it is possible to predict each Fib generation if (i) we have access to the previous generation of the one we want to reconstruct, or (ii) if we have access to two successive generations. This is possible since, as highlighted above, Fib can be defined as recurrence relation, where each generation is concatenated to the others, as expressed by the formula G_n_ = G_n-1_^G_n-2_ where ^ indicates concatenation [40,41]. This is a non-trivial property which is essential to be aware of in order to make predictions about the symbols that come up in the string at any juncture. Take, for instance, the ambiguity point determined by the sequence [01**01**]: despite the ambiguity of the last [1], we can easily predict that the sequence will be followed by a [1], under the condition that we are able to reconstruct the underlying derivation accessing the previous generation (g_n-1_), as exemplified in Fig 3.

We know that [1] in g_n_ can only result from rewriting a [1] in g_n-1_, and that the longer sequence [0101] in g_n_ can only result from rewriting a [11]-sequence in g_n-1_. Given that [111] is ungrammatical, it is possible to predict that [0101] cannot be followed by a [0], which would require an illegitimate third 1 in g_n-1_, but only by a [1], as exemplified in Fig 3. Resolving the ambiguity point determined by the sequence [0101**?**] as [1] is thus possible, under the condition that we consider the derivation, by accessing the previous generation.

**Fig 4:**
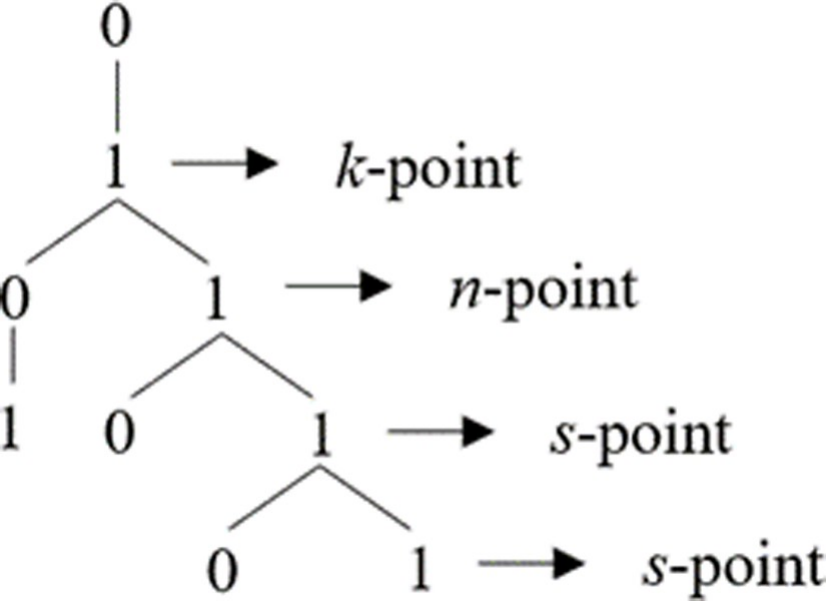
Structural representation of *k*-points, *n*-points and *s*-points.

Going back one generation is however not sufficient to disambiguate [1] in [011**01**]. This case is ambiguous in linear terms, in the sense that it can be followed either by a [0] or by [1] as per the Third Law. However, as we will show below, it is still possible to resolve this ambiguity and to predict which item will follow in each such context. In order to do it, though, we need to move one more step forward, accessing the hierarchical structure of the grammar. More precisely, we need to capitalise on the structure-based fact that not all [1]s are formally the same in Fib. In the growth of the Fib grammar, we can identify, indeed, three distinct types of [1]s with different structural properties as defined in the geometry of Fib, which we will dub *k*-points, *n*-points and *s*-points, following [40,41], and which can be informally defined as in (3) and represented as in Fig 4.

(3) a. A *k*-point is a [1] immediately dominated by a [0] and immediately dominating a [0 1]
  b. A *n*-point is a [1] immediately dominated by a *k*-point
  c. A *s*-point is a [1] immediately dominated by a *n*-point or by a *s-*point

**Fig 5:**
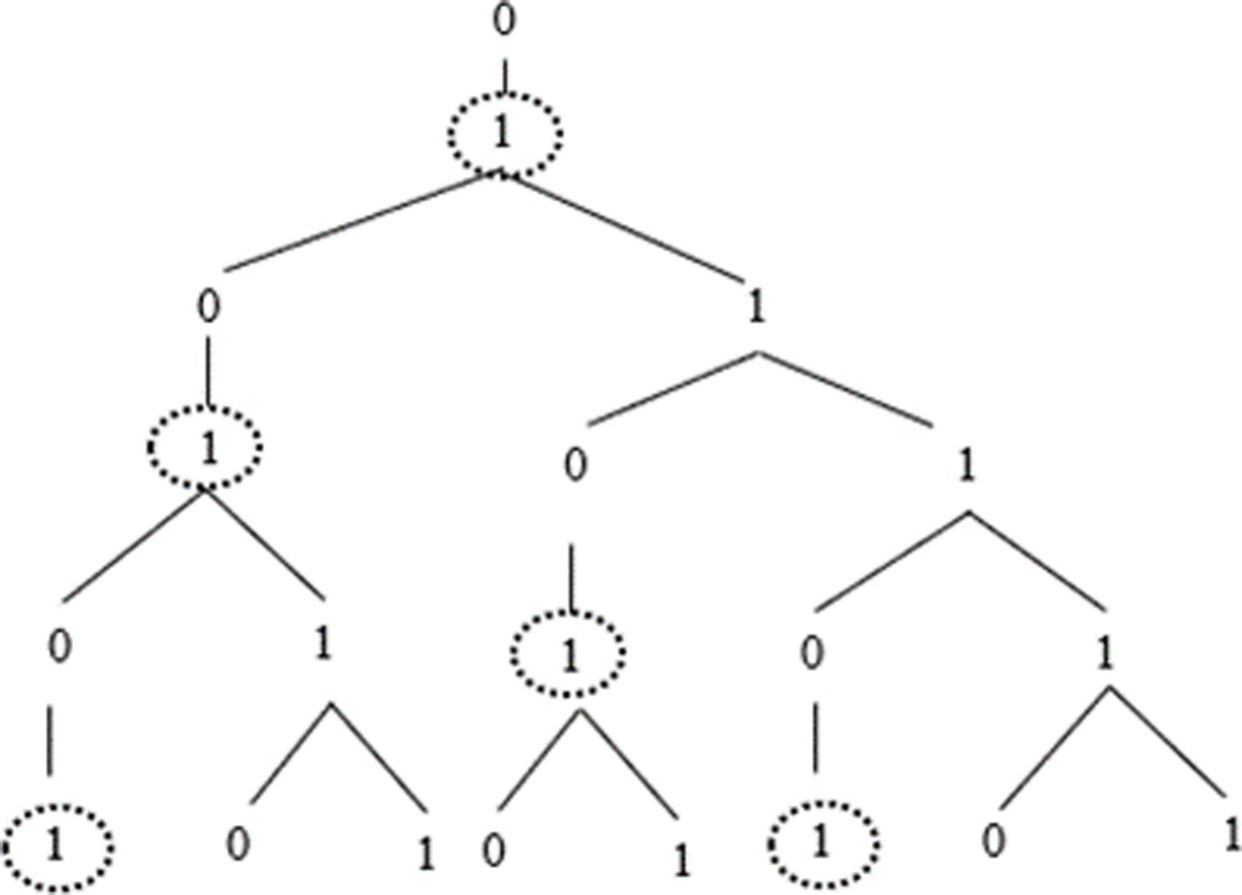
Graphical representation of *k*-points (circled with dotted line) in a Fib-derivation.

For the purposes of this paper, we focus only on *k*-points, which, as we will see, are the crucial elements that permit us to access the hierarchical structure of the grammar (for more details on *n*-points and *s*-points see [40]). Crucially, *k*-points can be defined only in specific L-grammars, all of which belong to the *asymmetric* class and not all of which are mutually equivalent [41]. As argued in (3a), *k*-points are defined as such on hierarchical grounds: they are immediately dominated by a [0] and they immediately dominate the constituent [1], as shown in Fig 5.

By identifying *k-*points in the string, we get information about three generations of the grammar: if we identify a *k*-point (g_n_), we are indeed able to build structure, that is to identify its mother node (a [0], g_n-1_) and its daughter nodes (a [0] and a [1], g_n+1_). Technically, we say that *k*-points provide information about their *neighbourhood* (i.e., the nodes a given point is immediately connected to). Furthermore, we know that the [1] in g_n+1_ is not a *k* itself (i.e. it is a *n*-point), since [1] is a natural constituent of the grammar resulting from the transition 1 → 01. Finally, if we consider the string of [0]s and [1]s produced by Fib, we must note that the last [1] in [11] is always a *k*-point, a regularity which is structurally significant, as we will see.

Another feature that must be noted concerns the fact that the succession of *k*-points in the string follows a specific pattern, which becomes completely predictable once access to the derivation has been gained. Being sensitive to *k*-points allows access to an abstract representation of the string: not just a sequence of [0]s and [1]s, but now a set of *k*s plus a specification of the distance between two adjacent *k*-points. We can exemplify this in (4) below, where generation 8 of Fib is first represented as a sequence of [0]s and [1]s with the *k*-points in bold, and then simply as set of *k*s and their distances, as in (5):

(4) 0 1**1** 01**1**0 1 0 1 **1** 0 1**1** 0 1 0 1**1** 0 1 0 1 **1** 0 1 **1** 0 1 0 1 **1**01
(5) _ _1_ _1_ _ _ _ 1_ _ 1_ _ _ _ 1 _ _ _ _ 1 _ _ 1 _ _ _ _ 1 _ _

Notice that, once we have learnt that the bold [1]s in (4) are *k*-points, we become able to reconstruct the string even without knowing if the other points are [0]s or [1]s. At this point, we can specify exactly what it means to characterise a [1] as a *k*-point (see also [40]):

- A *k* is always the daughter of [0]
- A *k* is always the mother of [0 1]
- A *k* will be either 3 or 5 symbols apart from the next *k*

It is crucial to note that only the first two structural regularities can be translated into rewriting rules since they make reference to *dominance* relations; the third one is of the same kind as the Third Law. Therefore, given a representation of a string like:

(6) 1 _ _ _ _ 1 _ _

the characterisation of what it means for a [1] to be a *k* allows for the reconstruction of that string. From left-to-right, we can proceed as follows (this effective procedure is a simplified version of the 2-tape FSA fully specified in [42]).

- There is a *k* at g_n_ → there has to be a [0] at *g*_n-1_ (or, in other words: there cannot be a [1] at *g*_*n-1*_ because *k*-points are never the daughter of another *k*-point)
- If there is a [0] at *g*_*n-1*,_ it means it must be followed by a [1] (as per the First Law). That means we can already fill the two following slots in *g*_*n*_: [0 1]:
  (6’) 1 **0 1** _ _ 1 _ _
- Because we know the metric imposed over the set of *k*-points, we know that there are two symbols before the next *k*-point. These must be [0 1], as no other option will be compatible with the abstract form in (6);
- The next symbol is a *k*-point, which means we can apply the two steps above again.

What we have done so far is to reconstruct the string [10101101] from a set of *k*-points and a specification of how far apart those *k-*points are.

We can now look at the distribution of *k*-points within a string: *k*-points may be 3 symbols apart or 5 symbols apart. Furthermore, these occurrences appear within structurally relevant units: [**1**01] and [01**1**01] (generations 3 and 4 of Fib). Because we have only these two options, we can keep track of the distribution of ‘close’ and ‘far’ distances. We can flesh this out: let K = {*k*_*1*_, *k*_*2*_, *…k*_*n*_} be the set of *k*-points in the infinite Fibonacci word. Then, the following regularities emerge:

- If *k*_*1*_ and *k*_*2*_ are 3 symbols apart, *k*_*3*_ will be 5 symbols apart from *k*_*2*_.
- If *k*_*1*_ and *k*_*2*_ are 5 symbols apart, *k*_*3*_ will be either 3 or 5 symbols apart from *k*_*2*._
- If *k*_*1*_ and *k*_*2*_ are 5 symbols apart and *k*_*2*_ and *k*_*3*_ are 5 symbols apart, *k*_*3*_ and *k*_4_ will be 3 symbols apart.

The regularities that emerge in the distribution of *k*-points mirror exactly the low-level local transition probabilities that govern the distribution of [0]s and [1]s in the Fibonacci string. This property, whereby regularities at different levels of abstraction follow the same pattern, will be referred to as *self-similarity*; the presence of self-similarity in the Fib derivation, we contend, is crucial to its learnability since it allows for hypotheses pertaining to local structure to be generalised. Another way of presenting this issue is to say that structural properties of Fib are *scale-free*: no matter how much we zoom in or out, the same structural patterns emerge. The notion of *scale* is essential both from a theoretical perspective and from an experimental perspective, and identifying properties of formal systems that remain the same across scales of organisation or levels of analysis (i.e., that are *scale-free*) is paramount to bridging the gap between computational, neurophysiological, and behavioral levels, which are defined at distinct levels of organisation and using distinct substantive primitives (see [38,43,44] for discussion).

Because the distribution of *k*-points follows the same regularities as the distribution of structural units (e.g., [101] and [01101], generations 3 and 4 in Fib) and even single symbols [0] and [1], it is easy to see that just like these, the distribution of *k*-points is *aperiodic* (although structurally governed): *k*-points do not arise every *n* items, and it is not possible to ‘count’ *n*-grams either since there is no repeating pattern. The distribution of *k*-points is given exclusively by the grammar, not determined in any way by low-level, string-based properties.

Summarising, successful prediction of the next symbol in a string depends on whether the parser can construct the local hierarchical structure of the derivation, an operation which is made possible by the identification of *k*-points: assessing sensitivity to these points represents then a tangible opportunity to tackle hierarchical learning as well as the interdependence between statistically-based linear learning and hierarchical learning in AGL in a much more direct and uncontroversial way than in previous studies.

Since this peculiar interdependence has never been tested so far, our principal aim is that of investigating whether individuals display a sensitivity to structure, measured as a function of the identification of *k*-points: in order to do so, we assessed implicit learning of a group of 10-year-old children administering a SRT task, specifically, a modified Simon Task in which the sequence of the stimuli followed the rules of two grammars which follow the same superficial transition laws but differ in constituent structure. In the first part we used Fib, whereas in the second part we employed Skip [40], another L-grammar which cannot be reduced to or expressed in terms of Fib. Fib and Skip have the same superficial regularities (i.e. the Three Laws), but their structural properties (i.e., its growth pattern, its internal constituents, its structural recurrences) are completely different as will be characterised below. Crucially for our purposes, a Skip grammar does *not feature k-points* due to its derivational properties: superficial regularities remain constant between Fib and Skip, but the backbone of structure is changed. The prediction is straightforward: if the process of learning an asymmetric grammar endowed with structurally significant elements which are identifiable through superficial regularities (here, Fib) may be expressed in developing or capitalising on sensitivity to these structurally salient points, the absence of these points should hinder learning even if other properties of the languages produced remain the same (e.g., the Three Laws).

### 1.2. Employing the Fibonacci grammar in AGL studies: Preliminary evidence

The structural template produced by Fib has been proposed to hold for varied aspects of natural language at different levels of linguistic analysis, including syllable structure and theme-rheme dynamics [45,46], metrical feet [47] and the X-bar schema for phrase structure [45,48,49]. In the present work, however, the interest in Fib is fuelled by its own properties, rather than by its relation to phrase-structure models of natural language syntax. In particular, we are interested in two properties of Fib in addition to the general characteristics that make L-systems different from systems in canonical form (lack of traffic convention and no distinction between terminal and nonterminal nodes): (i) self-similarity, as a consequence of the recurrence relation that lies at the foundations of the grammar, and (ii) asymmetric growth [32, 41]. These properties, when considered in the context of the debate between linear and hierarchical learning, make Fib a particularly good tool that can be exploited to address recursive processing also in AGL studies [50], since it permits to investigate the participant’s abilities to implicitly learn hidden regularities in a continuous string of symbols, while crucially disentangling the effects of statistically-based sequential learning from those of hierarchy-based learning in the absence of linguistic information (lexical semantics, prosody, etc.).

Some preliminary evidence for the robustness of implicit learning tasks featuring Fib has already been provided: in a pivotal research, [50,51] found that humans can discriminate between samples of true Fibonacci sequences and samples of foil sequences produced by means of *n*-gram substitution, up to substitution units of lengths 5 and 8; [52] found that participants were able to discriminate sequences of synthesised syllables following the Fibonacci grammar from random sequences with distributional properties matched to Fib. More recently, [53] found evidence of implicit learning in 10-year-old children (monolingual and bilingual children, with and without dyslexia), administering a modified Simon task developed following Fib’s Three Laws. As in traditional Simon tasks, children were simply asked to press 1 (i.e. a key on the left side of the keyboard) when they saw a red square and 0 (i.e. a key on the right side of the keyboard) when they saw a blue square, ignoring their position on the screen. Although group differences were found, with bilinguals being generally faster and dyslexics generally less accurate, all children showed evidence of having learnt both the First and the Second Law, displaying shorter reaction times as the task (composed by 432 trials) progressed. In particular, the fact that a red is always followed by a blue (i.e. that 0 is always followed by 1, *as per* the First Law) was acquired quite fast, after the exposure to approximately 144 items of the Fib-string, whereas that two blues are followed by a red (i.e. that 11 is always followed by 0, *as per* the Second Law) was acquired later, after approximately 288 trials. Importantly, responses to unambiguous trials were always faster and more accurate than those to ambiguous trials, confirming that the shorter latencies were really related to the learning of the relevant regularities, and not to a general effect of habituation to the task.

The properties of Fib render it an ideal candidate to assess both sequential learning, i.e. mastering of the First and the Second Law, as well as hierarchical learning, i.e. sensitivity to *k* points. Since the latter has not be studied yet, we decided to compare Fib to another grammar, Skip, which will be presented in detail in the Method section. Importantly, Skip shares the same surface regularities as Fib (the ‘Three Laws’), thus providing identical opportunities for sequential learning, but, crucially, it does not allow hierarchical reconstruction as in Fib, as there are no *k*-points. In other words, the sequence [011] is present in both grammars, but the second [1], a *k*-point in Fib, has no structural importance in Skip.

The hypothesis that emerges from these considerations is straightforward: if participants are sensitive to structure, i.e. if they are sensitive to *k*-points in Fib, they should thus behave differently in Fib and in Skip in correspondence to the [1] following the sequence [01].

### 1.3. Research questions and predictions

In light of what argued above, our study was designed to address specific research questions. First, we wanted to verify whether participants learnt the First and the Second Law outlined in (2) above: (i) the fact that a [0] is always followed by a [1] and (ii) the fact that the bigram [11] is always followed by a [0], even with a relatively low number of stimuli with respect to the study by [53]. Since these superficial regularities hold for both Fib and Skip, no differences were expected between the two grammars.

Besides this, we aimed at disentangling sequential from hierarchical learning, by verifying whether participants reacted differently to the sequence [01**1**] in Fib and in Skip. Since the second [1] is a *k*-point only in Fib, we expected reaction times to be faster in Fib in correspondence to [011] than in Skip, where the second [1] has no structural relevance.

## 2. Method

### 2.1. Participants

Our experimental protocol was administered to 22 children (14 females and 8 males) attending to Grades 3, 4 and 5 of the same public school in Spiazzo (Trento, North-East of Italy). Their age ranged from 8 to 11 years old (mean age 9.32, *SD* = 0.82). They were all native speakers of Italian and, as reported by parents and teachers, they had no cognitive or physical disorders, or language or learning difficulties. In addition, in order to have a measure of their nonverbal intelligence and to exclude the presence of children with possible cognitive deficits, we administered the CPM Raven [54]; all children scored within the normal range for their age.

Children were recruited through the schools they were in attendance at, after having received written informed consent from parents; no monetary compensation was provided to participants. The study was approved by the local ethics committee (Department of Neurological, Biomedicine and Movement Sciences, University of Verona) and conducted in accordance with the standards specified in the 2013 Declaration of Helsinki.

### 2.2. Materials

All participants were administered a modified Simon Task, which will be described below. The experiment was run on an Acer 15.6’ laptop using DMDX Automode version 6.0.0.4 software. The stimuli were four squares (dimensions 1012x536 pixels, BMP files) each for one of the four conditions. Each trial started with a fixation cross which appeared in the middle of the screen and remained visible for 500 ms and which was followed by a red or a blue square, either on the left or on the right side of the screen. As in traditional Simon tasks, participants were instructed to press a key on keyboard as they saw a square appearing on the screen of the laptop depending on their colour: if it was red, they had to press the number key 1, whereas if it was blue, that had to press the number key 0. Importantly, they were told that both red and blue squares could appear either on the left or on the right side of the screen, and that they had to ignore the position of the square on the screen, while paying attention only to its colour. There were four experimental conditions: blue congruent, blue incongruent, red congruent and red incongruent.

Participants had 1000 milliseconds to press a key: if they did not provide an answer within this time window, the following item was shown. The timing started with the onset of the item and ended with the response of the subject. Both accuracy and RTs data were collected.

In contrast to the traditional Simon task, our modified version, as in [53], presents two major changes, concerning (i) the sequence of blue and red squares and (ii) the occurrence of the incongruent trials.

As for the first aspect, the sequence of the coloured squares was not random, but entirely determined by an underlying grammar (Fib or Skip). More particularly, in the present version the task comprised 330 trials, divided in four blocks: the first three blocks, consisting of 89, 89 and 55 items each, for a total of 233 trials, corresponded to a full generation of the Fibonacci grammar (generation 12). The fourth block, instead, was composed by 97 items produced by the Skip grammar (generation 4).

Skip is obtained by means of a simple manipulation of Fib, which we refer to as an ‘expansion’. The general format for Fib is simply:

*g*_0_ → *g*_*1*_
*g*_*1*_ → *g*_*2*_

The manipulation of Fib we have used exploits the recurrence nature of L-systems. We generalise the schema above, such that:

*g*_*n*_ → *g*_*n+1*_
*g*_*n+1*_ → *g*_*n+2*_

In Skip, [0] rewrites as a generation which it does not immediately dominate in Fib (which would be [1]), but one which it *transitively* dominates. Thus, we formulate the following rewrite rule:

0 → 01 (*g*_2_)

And we proceed in the same way with [1]: we change the right-hand side of the rule to a generation of Fib that [1] transitively dominates:

1 → 01101 (*g*_*4*_)

**Fig 6:**
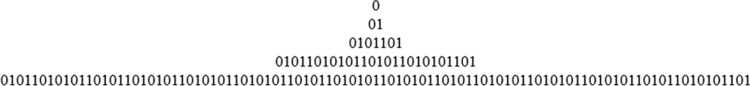
Graphical representation of a skip derivation.

Because [0] rewrites as Fib-g_2_ and [1] rewrites as Fib-*g*_4_, that is, as two *non-subsequent generations*, we refer to this as a *Skip* grammar (0 → 01; 1 → 01101, see Fig 6).

As can be noticed also by observing Fig 6, the transitional regularities established by Fib’s First and Second Law are respected, since a [0] is always followed by a [1] and two [1]s are always followed by a [0]. However, a fundamental property of Skip is that the elements in the alphabet [0] and [1] rewrite as two generations of Fib which are not subsequent (whence the name Skip); this property is in stark contrast to the *recurrence relation* that is at the heart of Fib. As a consequence, the hierarchical constituent structure of Skip is distinct from that of Fib, since it is not based on a recurrence relation between *g*_*n*_ and *g*_*n+1*_ for all values of *n*. Specifically, there are no *k*-points in Skip, as there is no point which is dominated by a [0] and that dominates a unit [01] which, recall, is the unit individualised by the First Law; a unit that has both *linear* and *hierarchical* significance. In Skip, the unit immediately dominated by [0] is [01], and each symbol in that sequence rewrites as well: it is no longer possible to rely on very local units to make the transition between linearity and hierarchy, in contrast to what is possible in Fib (see the Discussion for more details about the relation between linearity and structure in Fib in terms of parsing strategies). Since, as discussed above, *k*-points are fundamental in order to access the structure of Fib, comparing Fib and Skip can provide an extraordinary opportunity to assess sensitivity to the structure. Both grammars, indeed, present the sequence [01], whose continuation is ambiguous in purely linear terms, as discussed above, although it becomes predictable in Fib if the structure is accessed: remind, indeed, that the second [1] in [01**1**] is a *k*-point in Fib, and that subjects should be able to predict the sequence of *k*-points if there is sensitivity to structure. The crucial observation here is that since the hierarchical structure of Skip is completely different from that of Fib, the second [1] in the [01**1**] will not have any specific structural value.

Analysing the subjects’ performance across the four blocks permitted us to assess the presence of learning effects, as a decrease in reaction times or an increase in accuracy rates, without any need to have a training phase, as in traditional AGL studies. Indeed, children were never explicitly made aware of the presence of an underlying grammar nor of the fact that the grammar changed. Their performance was simply monitored throughout the task to check for block differences related to the implicit learning of the underlying grammar(s).

As discussed above, Fib and Skip share the same surface regularities, which, converted into colours, are shown below:

(7) Red → Blue
  Blue → Red Blue

These rules determine the transitional regularities that we repeat here for the reader’s convenience:

- First Law: A red is always followed by a blue (a sequence of two reds is ungrammatical)
- Second Law: Two blues are always followed by a red (a sequence of two blues is ungrammatical)
- Third Law: A blue can be followed either by a red or by a blue (the sequences [blue-red] and [blue-blue] are both grammatical)

Although these transitional regularities are identical in Fib and Skip, with opportunities of sequential learning being identical across the two grammars, hierarchical reconstruction of *k*-points is allowed only in Fib. The blue after [red-blue], indeed, is a *k*-point in Fib (i.e. it is dominated by a red, and it dominates another [red-blue] sequence), whereas it does not have any structural relevance in Skip.

The four blocks of items were presented sequentially without any interruptions between them and without the subjects explicitly noticing it.

Finally, the second modification of our task was that, consistent with the traditional Simon task, there were also incongruent trials, which occurred at regular intervals every sixth item, as in [53], with the aim of reducing the conflict with the congruent trials, by making incongruent trials statistically predictable. The presence of the incongruent items is however not directly relevant for the purposes of our study: they just contributed to keep the task engaging for the participants, avoiding boredom. Presenting them at a regular (and not too short) interval limited the effort required to inhibit the tendency to press the key on the same side of the item appeared on the screen [53].

There were 8 random practice trials in which subjects received feedback; after this short training, they had the chance to ask questions before the experiment began.

For the purposes of our study and consistently with the research questions outlined above, we were interested in verifying (i) whether the first regularity was learnt (i.e. a red is followed by a blue; see Analysis 1, in which we compared RTs and accuracy in each red trial following a blue one throughout the task), (ii) whether the second regularity was learnt (i.e. two blues are followed by a red; see Analysis 2, in which we compared RTs and accuracy in each red trial following a sequence of two blues) and (iii) whether participants displayed a sensitivity to structure, showing a different performance with the blues following [red-blue] in Fib (i.e. *k*-points) and in Skip (i.e. not-*k* points; see Analysis 3, in which we compared blue trials following a red-blue sequence in the three Fib blocks and in the Skip block). In the light of what argued above, no differences were thus expected between the two grammars in the learning of the two transitional regularities, whereas sensitivity to *k*-points was predicted to arise only with Fib and not in Skip.

### 2.3. Procedure

All children were tested individually in a quiet room by the third author. They were administered the CPM Raven task, followed by the modified Simon task. The Simon task lasted approximately 10–15 minutes, with a short (facultative) break after the end of the second block. The whole experimental session lasted approximately 30 minutes (10–15 minutes for the preliminary task and 10–15 minutes for the modified Simon task). After a week, when all children had been tested, they were asked whether they had noticed a pattern or a regularity in the sequence of items; none of them reported evidence for that.

## 3. Results

The children’s performance in the modified Simon task was analysed considering both reaction times (RTs) and accuracy rates. RTs were calculated only for correct answers, representing 91.07% of the responses. As outlined above, there was a time limit for participants’ responses, since the items disappeared after 1000 ms if no key was pressed. We then calculated the mean RT of each participant in each of the conditions tested. The results of three analyses that we ran to assess the presence of learning effects will be presented in the following sections; all statistical analyses were performed using SPSS v.21.0 (IBM Corp., Armonk, NY, USA). Since accuracy rates turned out to be at ceiling, especially for congruent trials, and almost constant across blocks, with no significant differences, we present here only the results of the statistical analyses concerning RTs.

### 3.1. Analysis 1: Blue trials following a red

To verify whether children learnt that a red item was always followed by a blue one we analysed responses to all congruent and incongruent blue trials following a red one, comparing RTs and accuracy rates of the participants across the four blocks of stimuli. As shown in Table 1, reporting mean RTs in each block for congruent and incongruent trials, a decrease in RTs can be noticed from Block 1 to Block 3, both in congruent and in incongruent trials, followed by a slight increase from Block 3 to Block 4.

**Table 1:** Mean (*Standard Deviation*) reaction times in each condition for each block for congruent and incongruent trials (Analysis 1).

	Block 1 (Fib)	Block 2 (Fib)	Block 3 (Fib)	Block 4 (Skip)
**Congruent trials**	503.80	484.62	445.31	458.27
	*91*.*30*	*67*.*60*	*59*.*71*	*64*.*18*
**Incongruent trials**	699.03	705.24	650.27	683.08
	*112*.*06*	*119*.*38*	*116*.*56*	*101*.*88*

Accuracy, instead, is at ceiling for congruent trials in each block (see Table 2), whereas it is lower for incongruent trials, slightly increasing in Block 3 and remaining stable between Blocks 3 and 4.

**Table 2:** Mean (*Standard Deviation*) accuracy in each condition for each block for congruent and incongruent trials (Analysis 1).

	Block 1 (Fib)	Block 2 (Fib)	Block 3 (Fib)	Block 4 (Skip)
**Congruent trials**	0.96	0.96	0.96	0.97
	*0*.*04*	*0*.*04*	*0*.*06*	*0*.*03*
**Incongruent trials**	0.71	0.71	0.78	0.76
	*0*.*24*	*0*.*25*	*0*.*20*	*0*.*22*

To verify whether these differences were significant, we ran a repeated-measures ANOVA with *Congruency* (Congruent vs. Incongruent trials) and *Block* (1, 2, 3 and 4) as within-subject variables.

As for RTs, we found a main effect of *Congruency* (*F*(1, 21) = 269.192, *p* < .001, partial η² = .928), indicating that incongruent trials were processed more slowly than congruent trials, and a main effect of *Block* (*F*(3, 63) = 4.688, *p* < .01, partial η² = .182), showing that reaction times decreased across blocks. The absence of a significant *Congruency × Block* interaction (*F*(3, 63) = .432, *p* = .731, partial η² = .020), indicated that RTs decrease across blocks similarly for congruent and incongruent trials. Post-hoc comparisons with Bonferroni correction revealed that, considering congruent and incongruent trials together, there was a marginally significant difference in RTs between Block 3 (*M* = 547.79, *SD* = 15.99) and Block 1 (*M* = 601.42, *SD* = 18.99, *p* = .051), and a significant difference between Block 4 (*M* = 570.67, *SD* = 14.82) and Block 1 (*p* < .05). Conversely, no differences were found between Blocks 1 and 2 (*M* = 594.93, *SD* = 17.05, *p* = 1.000) and between Blocks 3 and 4 (*p* = 1.000).

Summarising, the decrease in RTs suggests that the First Law (i.e. a red is always followed by a blue) was learnt by children by Block 3 (approximately after 178 items). Notice that, as argued above, this regularity, which can be processed by a low-level statistical process reflecting learning linear regularities, is identical in Fib and in Skip. This result is compatible with the one obtained by [53], where this regularity was learnt by children of the same age in correspondence between Block 1 and Block 2, that is approximately after 144 items. The fact that incongruent trials are processed less accurately and more slowly than congruent ones, instead, is not surprising and should be simply interpreted as a side-effect of the Simon task, in which incongruent items are more difficult to process due to the interference between the position of the square on the screen and the key to be pressed. However, it is interesting to notice that there was no interaction between block and congruency, indicating that participants could predict the colour of the upcoming square both with congruent and incongruent trials.

### 3.2. Analysis 2: Red trials following two blues

After having verified that the first regularity was learnt, we aimed at assessing the presence of improvements related to the learning of the second regularity, i.e. two blues are followed by a red. To this aim, we analysed the responses to all congruent and incongruent red trials following two blues, comparing RTs and accuracy of the participants across the four blocks of stimuli, as shown in Table 3, reporting mean RTs and accuracy rates in each block.

**Table 3:** Mean (*Standard Deviation*) reaction times for congruent and incongruent trials in each block (Analysis 2).

	Block 1 (Fib)	Block 2 (Fib)	Block 3 (Fib)	Block 4 (Skip)
**Congruent trials**	546.11	576.46	533.39	556.06
	*(63*.*48)*	*(59*.*71)*	*(69*.*40)*	*(81*.*23)*
**Incongruent trials**	642.15	654.71	625.30	670.56
	*(90*.*32)*	*(80*.*20)*	*(82*.*75)*	*(156*.*44)*

RTs seem to be almost constant across blocks, as well as accuracy, which is at ceiling for congruent trials in each block (see Table 4), whereas it is lower for incongruent trials, slightly increasing in Block 3 and remaining stable between Blocks 3 and 4.

**Table 4:** Mean (*Standard Deviation*) accuracy in each condition for each block for congruent and incongruent trials (Analysis 2).

	Block 1 (Fib)	Block 2 (Fib)	Block 3 (Fib)	Block 4 (Skip)
**Congruent trials**	0.96	0.94	0.95	0.92
	*0*.*06*	*0*.*07*	*0*.*07*	*0*.*10*
**Incongruent trials**	0.88	0.86	0.93	0.82
	*0*.*16*	*0*.*18*	*0*.*17*	*0*.*24*

To verify whether these differences were significant, we ran a repeated-measures ANOVA with *Congruency* (Congruent vs. Incongruent trials) and *Block* (1, 2, 3 and 4) as within-subject variables.

As for RTs, we found a main effect of *Congruency* (*F*(1, 21) = 109.440, *p* < .001, partial η² = .839), indicating that incongruent trials were processed more slowly than congruent trials, but no effect of *Block* (*F*(3, 63) = 1.945, *p* = .131, partial η² = .085), nor *Congruency × Block* interaction (*F*(3, 63) = .584, *p* = .627, partial η² = .027). This suggests that the second regularity was not learnt, neither for congruent nor for incongruent trials.

Summarising, differently from the first regularity, in this case we did not find any learning effect, either in terms of RTs or accuracy, as testified by the absence of block effects, indicating no improvements throughout the task and therefore no evidence of learning. Notice that this result is not completely incompatible with the one reported by [53], who found that children learnt this regularity after 288 items: a possible explanation for the lack of learning of the Second Law in the current study lies in the limited number of items administered. We predict indeed that also this regularity would be learnt with a sufficient number of trials, while leaving this issue to future research.

### 3.3. Analysis 3: Sensitivity to hierarchical structure

As argued above, *k*-points, which can only be identified through a hierarchical reconstruction of the grammar, are present only in Fib. To verify whether participants were sensitive to the presence of *k*-points in Fib, we compared RTs and accuracy in correspondence of all blue trials following a [red-blue] sequence in Fib and in Skip. As in previous analyses, both congruent and incongruent trials were considered. Since there was only a limited number of *k*-points in each of the three Fib blocks, especially concerning the incongruent trials, we decided to consider the three blocks together in order to compare the sensitivity to *k*-points in the two grammars.

As shown in Table 5, RTs are longer in Skip than in Fib, both in congruent, and, even more markedly, in incongruent trials.

**Table 5:** Mean (*Standard Deviation*) reaction times for congruent and incongruent trials in Fib and in Skip (Analysis 3).

	Fib (Blocks 1, 2, 3)	Skip (Block 4)
**Congruent trials**	532.07	542.39
	*63*.*62*	*67*.*81*
**Incongruent trials**	650.48	701.52
	*100*.*79*	*117*.*78*

Accuracy, instead, is at ceiling in congruent trials, whereas it is much lower in incongruent trials, and in particular in the Skip block (see Table 6).

**Table 6:** Mean (*Standard Deviation*) accuracy for congruent and incongruent trials in Fib and in Skip (Analysis 3).

	Fib (Blocks 1, 2, 3)	Skip (Block 4)
**Congruent trials**	0.94	0.96
	*0*.*04*	*0*.*07*
**Incongruent trials**	0.61	0.50
	*0*.*20*	*0*.*29*

To verify whether these differences were significant, we ran a repeated-measures ANOVA with *Congruency* (Congruent vs. Incongruent trials) and *Type of Grammar* (Fib vs. Skip) as within-subject variables.

As for RTs, we found a main effect of *Congruency* (*F*(1, 19) = 108.623, *p* < .001, partial η² = .851), indicating that incongruent trials were processed more slowly than congruent trials, and a main effect of *Type of Grammar* (*F*(3, 63) = 4.806, *p* < .05, partial η² = .202), showing that reaction times were faster in Fib (*M* = 591.28, *SD* = 16.80) than in Skip (*M* = 621.95, *SD* = 18.61). The absence of a significant *Congruency × Block* interaction (*F*(1, 19) = 2.140, *p* = .160, partial η² = .101), indicated that with both congruent and incongruent trials RTs were faster in Fib than in Skip.

Summarising, a significant difference was found in how participants reacted to blue trials occurring after a [red-blue] sequence in the two grammars: RTs, indeed, were faster, in both congruent and incongruent trials, in Fib than in Skip, indicating that children could effectively exploit the cues provided by hierarchical structure in predicting the occurrence of *k*-points. As in the previous analyses, the effect of learning was visible only in RTs and not in accuracy. However, it is worth noticing that, differently from Analysis 1 and 2, accuracy, despite being at ceiling for congruent trials, was markedly low, approaching chance level, in incongruent trials. This effect is arguably related to the fact that in previous conditions, the occurrence of a blue trial after a red one, or of a red trial after two blue ones, could be effectively predicted. This was not possible, instead, in Skip, where the [red-blue] sequence was completely ambiguous and could be followed either by a red or by a blue.

## 4. Discussion

Our goal in this experiment was to assess implicit learning by means of a SRT task featuring the Fibonacci grammar, which was administered to 22 10-year-old typically developing children with a twofold purpose. We aimed, on the one hand, at testing the acquisition of linear regularities, (i) a red is always followed by a blue (Analysis 1), and (ii) two blues are always followed by a red (Analysis 2) and on the other hand, at determining whether participants showed evidence of sensitivity to structure, displaying different performance in blue trials following a [red-blue] sequence in Fib (where the target blue trials correspond to structure-sensitive *k*-points) and in Skip (where they do not, Analysis 3).

**Fig 7:**
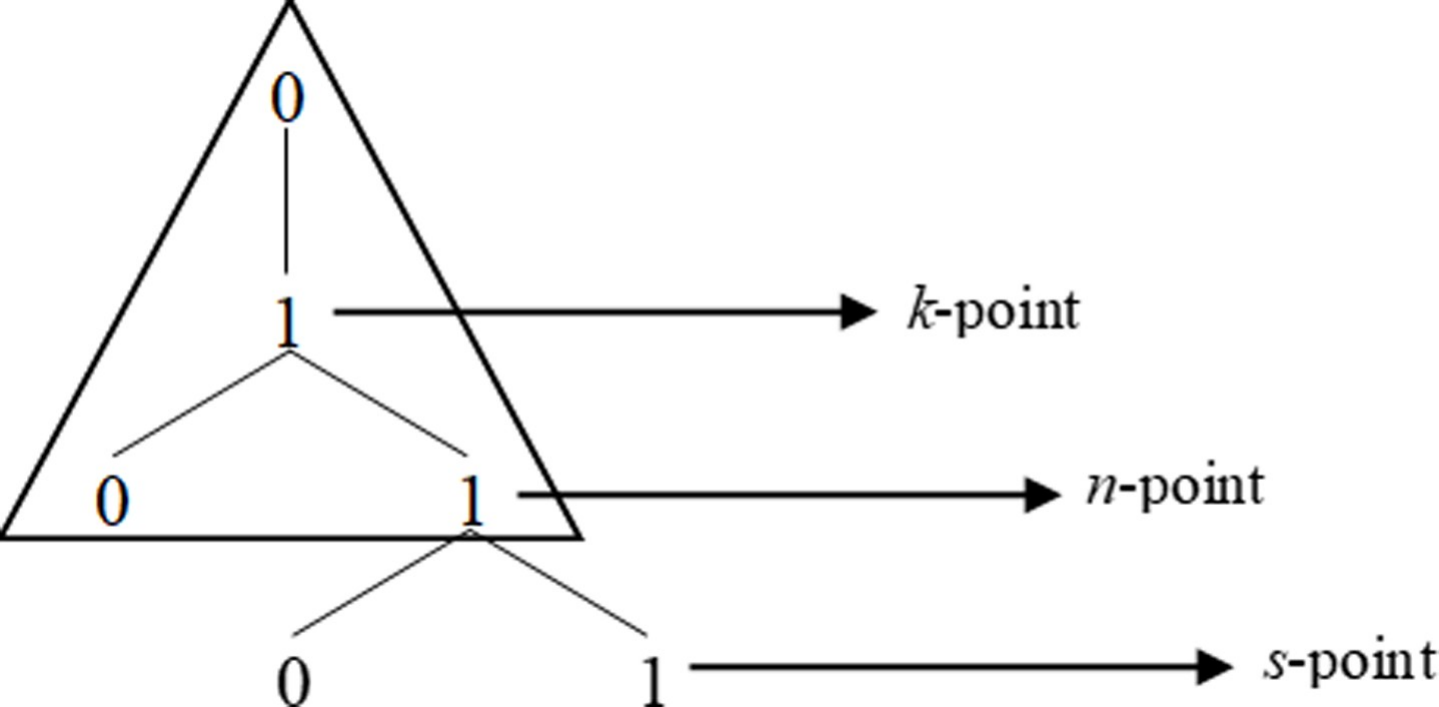
Representation of the configuration of *k*-, *n*- and *s*-points in Fib.

These two perspectives, linearly and structurally based, are connected by the fact that in Fib the First Law allows us to identify *k*-points and therefore to get access to the basic structural block which is recursively used to tessellate the space defined by the grammar: if every [0] is followed by a [1], there is a statistical generalisation that is robust enough (as it gets reinforced the more exposure to the signal a parser has) to support a parsing hypothesis whereby [01] units are grouped. As observed above (Section 1), a segmentation of the string attending to the First Law leaves some [1]s alone: these are *k*-points. As observed in [40], it is possible to formulate an effective procedure to yield these results: in Fib, a segmentation of the string *as per* the First Law (which, recall, is a generalisation over transition probabilities between trials) isolates points which are structurally relevant (i.e., *k*-points). Importantly, the regularity expressed by the First Law is reliable enough so that in information-theoretic terms, there is reinforcement of the signal and that allows for generalisations in the form of parsing hypotheses. In turn, identifying and keeping track of *k*-points allows a parser to disambiguate all linear conditions: there is an interaction between linear relations which bootstrap structure and structural regularities (the rhythm of *k*-points) which allows for the formulation of reliable hypotheses about what symbol will come next at any juncture. This is so because, derivationally, *k*-points are labels for their structural neighbourhood: identifying a point as a *k*-point entails information about the structural context of that point, its parent node and its daughter nodes, as discussed above, and represented in the Fig 7 below:

As explained in Section 1, the distribution of *k*-points is *not periodic*: no mechanical counting process at the string level (i.e., no operation targeting *n-*grams) can predict where *k*-points will occur. The identification of *k*-points, in summary, derives from a process of *segmentation* of an output string from the Fib grammar into [01] units and *labelling* of these units; a process that is foundational to both information-theoretic and structurally-based parsing models (see [55–58] among many others; [59] for further discussion). In this case, and differently from other AGL studies mentioned above, we can thus firmly exclude the possibility that the demonstration of sensitivity to structure can be reduced to or expressed in the form of counting or subitising mechanisms.

**Fig 8:**
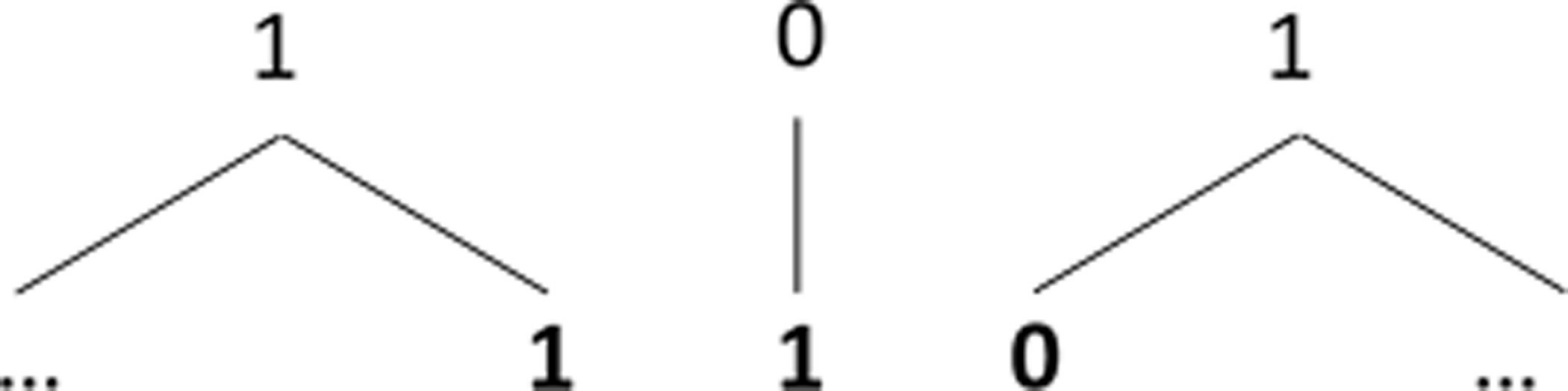
Representation of the trigram [110] in Fib.

The results of Analysis 1 suggest that, in consonance with previous findings [53], the First Law is indeed acquired: participants become sensitive to the distributional fact that every red is followed by a blue, regardless of congruency. This is the simplest regularity in Fib, but one that is essential in order to allow the transition into hierarchical computation: acquiring the First Law is a necessary condition to crack the hierarchical structure of the Fib grammar open given the relation between precedence and dominance that is specific of this asymmetric L-system. The Second Law, in contrast, is a regularity that has no structural import: there is nothing in [110] (or blue-blue-red) that corresponds to derivational properties of the grammar. As a matter of fact, [110] breaks up constituency, as shown in Fig 8:

Analysis 2 shows no learning effect in the number of trials used in this protocol, in contrast with earlier work [53] showing that with a larger number of trials the Second Law is indeed learnt. However, it is important to note the fact that the Second Law is acquired in a way orthogonal to structural sensitivity: as argued above, there is no hierarchical payoff to keeping track of this regularity, which may render it less “urgent” to acquire. On the other hand, this is certainly not incompatible with the experimental finding that subjects, having enough time, become sensitive to it.

Even with a reduced number of trials with respect to previous experiments, Analysis 3 showed a difference between Fib and Skip at crucial hierarchical junctures: the last [1] in [01**1**], which corresponds to a *k*-point in Fib but not in Skip. Both grammars produce strings where [011] is found, and in both cases the density of the *n*-gram (calculated as *total number of symbols in the string/number of occurrences of the n-gram*) defines a convergent series. *Prima facie*, from a strict string-based information-theoretic approach, there should be no difference between the Fib condition and the Skip condition in what pertains to [011]. This is crucial: in both languages (the one produced by the Fib grammar, and the one produced by the Skip grammar) the Three Laws hold, and in both the 3-gram [011] is found; the only differences between Fib and Skip are *derivational*, since Skip was obtained by means of a manipulation in the rules of Fib that takes into consideration relations between *generations* of the grammar. As highlighted above, there are no *k*-points in Skip; another way of saying this is that the last [1] in [011] in Skip is a completely unremarkable [1], both in terms of linearity and hierarchy. If parsing is guided by structural principles *as well as* sensitivity to string-based transition regularities, however, the difference between Fib and Skip can be accounted for: in Fib, identifying (indexing) the last [1] in [011] comes with a big structural payoff. We are led therefore to a new version of an old question–*how do humans manage to extract structure embedded in strings of stimuli*? We have observed that if we follow the simple transition probabilities in the Fib generations we can chunk the string into [01] units (which are fully predictable bi-grams, as per the First Law), with stranded [1]s left over (see [42]). Those [1]s are always *k*-points, but we only know that by reference to our knowledge of the derivation and its growth properties (*k-*points being originally defined in terms of the intrinsic geometry of the Fib grammar [41])–this information is not accessible to the participant. The subject, insofar as it behaves as if it were sensitive to these nodes in the derivation, is going beyond the information available in the surface statistical distribution of symbols in the string. How? What constitutes the additional step? We can be more specific about the nature and consequences of this step, getting into issues that we intend to fully develop in future research.

Under a structurally-sensitive parsing, whereby the First Law yields a consistent *segmentation* of the string and these segments are *labelled* (i.e., taken as a unit for purposes of further computation), the 3-gram [011] receives a structural analysis [[01][1]]; this is the key step towards structural reconstruction insofar as the neighbourhoods of *k*-points provide a partial tessellation of the space defined by Fib. This is a particular aspect of Fib expansions and can be described as a condition of *symmetry*—the linear order within [01] (0 precedes 1) is mirrored by the derivational order associated with the rules of the grammar: [0] generates (rewrites as) [1] and *then* [1] generates [01]. Thus, we can propose that the human parser infers that the sequence 01 is in fact the constituent [01] because of the deterministic transition from 0 to 1 at the linear level (the First Law), and gives it a label from the alphabet. This is the familiar *is-a* relation and constitutes the first step in the moving from linear order to hierarchy. Chunking of this type makes the recognition task easier for the subject. Further chunking leverages the aperiodic regular properties–if [01] is a chunk then stray [1]s are, too. The condition of symmetry (i.e. linear order is isomorphic to dominance) is consistent with this strategy and consequent symbols in the string are reliably predicted reinforcing the subject’s parsing hypotheses. Symmetry and chunking are happy bedfellows for Fib: in Fib, every [01] is labelled as [1] and every [1] following [01] is labelled as [0].

The situation in Skip is drastically different. The structural analysis of [011] that in Fib opens the gate to structure reconstruction through self-similarity (as explained in Section 1), provides no structural information whatsoever in Skip. In this state of affairs, the differences in RTs between Fib and Skip found in Analysis 3 can be accounted for: in order to react faster to the last [1] in [011], subjects must not only acquire the First Law, but also become aware of the structural relevance of these points, such that keeping track of them is worth the effort.

Summarising, this study provides far-reaching results: on the one side, we found evidence for implicit learning administering an AGL task that remarkably differs from previous studies, crucially overcoming their major weaknesses: real implicitness of the task and opportunity to disentangle sequential and hierarchical learning. Employing a SRT task, we found that the First Law, which is essential to access the hierarchical structure of the grammar, was successfully acquired by children, whereas the Second Law was not learnt, presumably related to the reduced number of trials of this task with respect to previous studies (this regularity was acquired between item 288 and item 432 in [53]’s study, whereas there were only 330 items in the present study) as well as to its lower structural importance, as discussed above. Even more importantly, perhaps, our results represent a new original proof for the existence of hierarchical learning, as testified by sensitivity to *k*-points in Fib and the way this sensitivity interacts with string-based statistical information.

## 5. Conclusion

In this study, we aimed at exploring sequential and hierarchical learning by means of an implicit learning protocol, the modified Simon Task, in which the sequence of the stimuli was determined by the rules of the Fibonacci grammar and its expansion, Skip. By administering a SRT task instead of asking for grammaticality judgments as typically done in AGL studies, we made sure that the implicitness of the task was preserved. Moreover, adopting L-systems instead of grammars in canonical forms allowed us to disentangle sequential from hierarchical learning in a more precise way, by comparing the participants’ performance in sequences which were superficially identical but structurally different in Fib and Skip (011, where the second [1] is a *k*-point, with a crucial structural importance in Fib but of no relevance in Skip). Importantly, we found evidence of both sequential learning (*as per* the First Law) and hierarchical learning, as shown by the sensitivity to *k-*points in Fib. One limitation of the present study consists in the limited number of stimuli of the Fib blocks, which was considerably lower than in the study conducted by [53]: this aspect can be considered responsible for the absence of learning of the second regularity, as well as for the need to collapse the three Fib blocks together in order to verify the sensitivity to *k*-points in Fib and Skip, preventing us to provide evidence for a real effect of *k*-points learning. We believe that this limitation should be addressed by future studies, developing a longer task which could allow to verify our hypotheses. In fact, some preliminary results of a study we are presently conducting employing four 89-items blocks of Fib show indeed significant block effects, providing evidence for learning of both the second transitional regularity and sensitivity to the structurally relevant *k*-points.

All in all, the results of the present experimental protocol strongly suggest that the parser humans apply in order to process strings produced by the Fibonacci grammar aims not only at assessing transitional probabilities, but also, crucially, at identifying structural patterns around specific points in the strings (which we have called *k*-points) that are made available due to the asymmetric format of this grammar. Exactly as we know that the parser must be endowed with the computational resources necessary to calculate transitional probabilities in the strings (the Three Laws discussed above), we should conclude that the parser must make use of the kind of computational resources that are necessary to act in a structure-sensitive working space, a space that may be accessed by virtue of a symmetric relation existing between precedence and dominance in local bi-grams. The questions that arise around a precise characterisation of this hierarchy-related resources, as well as around possible constraints in the way these resources are deployed in the parser, open new exciting avenues for future research.

## Supporting information

S1 Dataset (XLSX)
